# Identification of preferred reminder systems and patient factors to promote adherence in the management of urinary incontinence

**DOI:** 10.1016/j.pecinn.2022.100067

**Published:** 2022-07-20

**Authors:** Nour Bundogji, Gina Toma, Aqsa Khan

**Affiliations:** a College of Medicine, University of Arizona College of Medicine Phoenix, Phoenix, AZ, USA; b Department of Urology, The Mayo Clinic in Scottsdale, Scottsdale, AZ, USA

**Keywords:** Urinary incontinence, Treatment adherence, Smartphone application

## Abstract

**Objective:**

To investigate comfort level and preferences of automated reminder systems (mail, email, text message, phone call, patient-portal message, and/or smartphone application) to promote adherence to recommended therapies for patients seeking care for urinary incontinence (UI) at our urology clinic in Phoenix, Arizona.

**Methods:**

Anonymous surveys were distributed in English to adult patients with UI from 4/2019–5/2019. Patient demographics, UI type, and access to and use of the Internet, smartphone and patient-portal were assessed. Using a Likert scale, patients indicated level of comfort with each reminder system and numerically ranked each system. Statistical analyses were performed to identify patient characteristics associated with reminder modality and determine significance in ranking of systems.

**Results:**

Fifty-seven patients (67.3 ± 16.3 years) completed the survey with an 87% response rate. Text-message and phone call reminder modalities were ranked the highest compared to other modalities (*p* < 0.05). A Chi-squared test showed no correlation between preferred reminder system modality and type of incontinence, age, gender, race/ethnicity, or language spoken (*p* > 0.05). Internet usage and access significantly correlated with preference for smartphone application and patient-portal message reminder systems (*p* < 0.05).

**Conclusion:**

Patients reported they were extremely comfortable with all communication modalities, except for smartphone applications in which patients were the least comfortable. The modalities most preferred by patients were phone call and text message and least preferred were patient portal and smart phone application. In conclusion, phone calls and text messages were the most preferred communication modality, with smart phone applications as the least comfortable.

**Innovation:**

This study demonstrates the potential utility of specific reminder modalities for patients seeking help with treatment adherence.

## Highlights

• UI is a common health condition where inadequate adherence to treatment can pose a risk for complications and hospitalizations
• This study is the first to explore patient preferences for reminder modalities to help promote treatment adherence for UI.
• Text messaging and phone call reminder modalities were most preferred in patients with UI.

## 1 Introduction

Urinary incontinence (UI), the involuntary loss of urine, is a common health condition that has been shown to decrease quality of life. The overall prevalence of UI among women 60 years or older in the US is estimated to be 14% [ 1]. Despite this high prevalence, only 25% of affected women seek care, and of those, less than half receive treatment [2]. Untreated incontinence is associated with falls and fractures, urinary tract infections, sleep disturbances, and depression [[3], [4], [5]].

The recommended first-line approach for urinary incontinence is conservative treatment and includes pelvic floor muscle exercises (PFMT), bladder retraining, and fluid modification. The success rates of PFMT in treating UI range from 60% to 75% [ [6], [7]]. It is essential for PFMT success that the patient correctly perform the exercise and adhere to the recommended treatment [8].

Although there is a high prevalence of UI, it remains underdiagnosed and undertreated [12]. Furthermore, long-term follow-up results are variable. In a systematic review of 19 studies on PFMT, adherence ranged from 10% to 70% [13]. Nonadherence is a complex human behavior representing a major risk factor in chronic conditions and has become a significant healthcare burden. Adherence rates in chronic disease management are multifactorial in origin with poor adherence reducing quality outcomes [14]. Additionally, adherence tends to decrease during long-term follow-up [15]. It has also been noted that the most common complaint about barriers to adherence with conservative therapies is “difficulty remembering to do the exercises” [16].

Thus, a strategy to remind patients may be helpful. Some studies have proposed and evaluated the use of technology to treat and follow urinary incontinence. Sjöström et al. evaluated an Internet-based treatment and concluded that the lack of face-to-face contact was not a disadvantage in terms of adherence in women using this technological approach [17]. Asklund et al. used a mobile device application and found relevant improvements in UI evaluated with questionnaires and improvement in adherence in the app group [18]. However, it is unclear which reminder modality is the most useful for this patient population.

Considering treatment adherence is often multifactorial, a multidimensional approach is required. Inadequate adherence to conservative treatment of UI in patients greater than 65 years old can pose a risk for complications and hospitalizations [9]. Current literature suggests the need to investigate the use of technology such as electronic-based resources to assist and maximize adherence [10].

Mobile health apps are a growing field that offers a range of possibilities for delivering health services that enable patients to increase treatment adherence, especially those with limited access to health care, including physical, financial, or geographic factors [11]. When SMS messages were used as reminders, they were highly effective in increasing adherence to prescription medication [[19], [20], [21], [22]]. Interestingly, it has been posited that SMS reminders may help influence memory neural circuits to promote behavior change toward adherence [[23], [24], [25]]. Many studies have proven the efficacy of different reminder forms - such as phone calls, emails, and short message services (SMS) [26]. Teeng et al. demonstrated that reminders through mobile messaging applications are effective in improving outpatient attendance and medication adherence among patients with depression [26]. In patients with tuberculosis, a systematic review found digital health technologies (DHT), including SMS reminders, effectively improved medication adherence in this patient population [27].

Although DHTs have been found to be effective in promoting treatment adherence in some populations, these systems do not come without their limitations. For instance, most commercial apps lack adequate security measures [28], leading to potentially unacceptable risks to patient confidentiality. Additionally, studies that assessed DHT-based interventions were under trial conditions, which may not represent the real-world condition for implementation. Although acceptance of an app positively influences its successful use in self-management [29,30], potential users were not consulted about their needs in the development of most of these apps [28,31]. Numerous factors may influence the effect of a DHT's intervention in promoting treatment adherence and include: population density, facilities, transportation, smartphone network coverage, health care systems, staff dependence, and cost-efficiency [32]. Thus, it is imperative that treatment reminder interventions consider these limitations prior to implementation.

Furthermore, healthcare professionals and patients may lack confidence and not consider these resources as part of routine care. Thus, reminder modalities such as phone call, text message, and/or patient portal messages need to be evaluated and compared with various population groups to identify the most patient-friendly method that will promote treatment adherence in the management of urinary incontinence. There is a lack of synthesized evidence from the literature on the effectiveness of a reminder system in promoting UI treatment adherence. The aim of this qualitative study is to investigate comfort level and preferences for a reminder system to help increase adherence to recommended therapies and to promote continence in adults with UI. Using a self-reported, anonymous survey, we aim to assess patient preferences for a treatment reminder system (email, text, phone call, patient portal message, and/or smart phone application). We will also determine specific patient demographic variables associated with each preference.

## 2 Methods

We conducted a prospective study from April 2019 to May 2019 at an outpatient urology clinic in a tertiary care hospital in Phoenix, Arizona. The study was granted exemption from the Institutional Review Board at Mayo Clinic Phoenix Campus.

### 2.1 Participants

Participants were adult patients 18 years or older and referred for evaluation of UI or diagnosed with UI and receiving treatment by a urologic specialist at the same care facility in Phoenix, Arizona.

### 2.2 Study design and procedures

Anonymous self-reported surveys were created by the research team with the intent to measure which automated treatment reminder system patients prefer. Surveys were administered by a student research trainee in English after the initial office visit to adult patients meeting inclusion criteria. The survey was not pilot tested prior to administration and assessed the following: age, gender, race/ethnicity, urinary incontinence type, access to internet, smartphone use, and patient portal access. Prior to survey completion, participants were asked to measure their level of comfort in a hypothetical treatment adherence program that would be implemented by an automated reminder system of their preference after their care visit. Likert scale was used to measure level of comfort with each reminder system from 1 to 5 with 1 being ‘least comfortable and 5 being ‘most comfortable (mail, email, text message, phone call, patient-portal message, and/or smartphone application). These systems were then ranked numerically from most to least preferred.

### 2.3 Statistical analysis

Statistical analyses were performed using SPSS software (IBM Corp. Released 2015. IBM SPSS Statistics for Mac, Version 23.0. Armonk, NY: IBM Corp.) Descriptive statistics and Spearman's rho were performed to identify patient characteristics associated with reminder modality preference. A Chi-squared test was used to assess the association between modality preference and patient demographics. All *p*-values were 2-sided and a value less than 0.05 was considered statistically significant.

## 3 Results

A total of 90 patients were approached for inclusion in this study. 57 adult patients (mean 67.3 ± 16.3 years) with UI completed the questionnaire and were included in the study. We excluded seven participants that consisted of two males and 5 females due to incomplete survey results. A recruitment rate of 87% was determined and all patients were seeking urologic care at the same urology clinic in Phoenix Arizona. Table 1 summarizes patient characteristics identified via the survey. Of these participants, 42% were classified as having urgency incontinence, while 14%, 32%, and 2% had stress, mixed, or other incontinence, respectively. The majority (94%) of the patients were female, white (84%), with a primary language of English (96%). Technology savviness was measured in the survey to evaluate participants' technology usage, accessibility and interest with the various treatment modalities listed in the survey. 90% of patients had access to the Internet, and 78% stated they use the Internet. 96% of patients had access to a smartphone, and 80% indicated use of a smartphone. 84% of patients indicated access to a patient-portal, and 68% indicated use of a patient-portal.

**Table 1:** Demographic table.

Variable Name	Mean (SD)/ %(N) Total (*n* = 50)
Age	67.27 (16.25)
Gender	
Male	6.0 (3)
Female	94.0 (47)
Race/Ethnicity	
White	84.0 (42)
Hispanic/Latino	6.0 (3)
American Indian	4.0 (2)
Asian	4.0 (2)
Other	2.0 (1)
Primary Language	
English	96.0 (48)
Spanish	1.0 (2)
Other	1.0 (2)
Secondary Language	
English	4.0 (2)
Spanish	8.0 (4)
None	88.0 (44)
UI Type	
Stress	14.0 (7)
Urgency	42.0 (21)
Mixed	32.0 (16)
Other	2.0 (1)
Internet Access	
Yes	90.0 (45)
No	10.0 (5)
Internet Use	
Yes	78.0 (39)
No	22.0 (11)
Smartphone Access	
Yes	96.0 (48)
No	4.0 (2)
Smartphone Use	
Yes	80.0 (40)
No	20.0 (10)
Patient-Portal Access	
Yes	84.0 (42)
No	16.0 (8)
Patient-Portal Use	
Yes	68.0 (34)
No	32.0 (16)

**Fig. 1:**
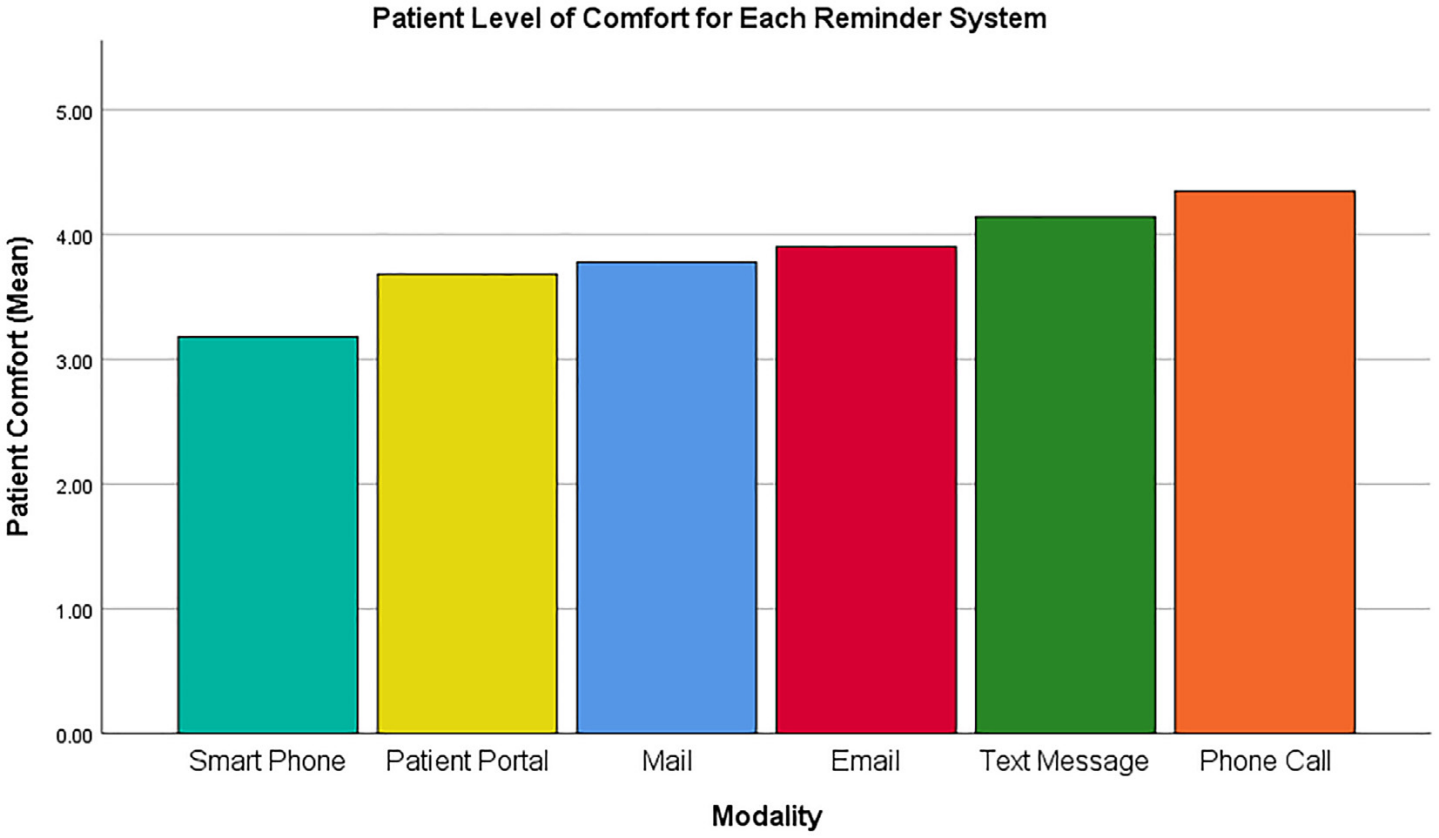
Mean patient-reported comfort level of each treatment reminder system modality (1.00 = least comfortable 5.00 = extremely comfortable).

**Fig. 2:**
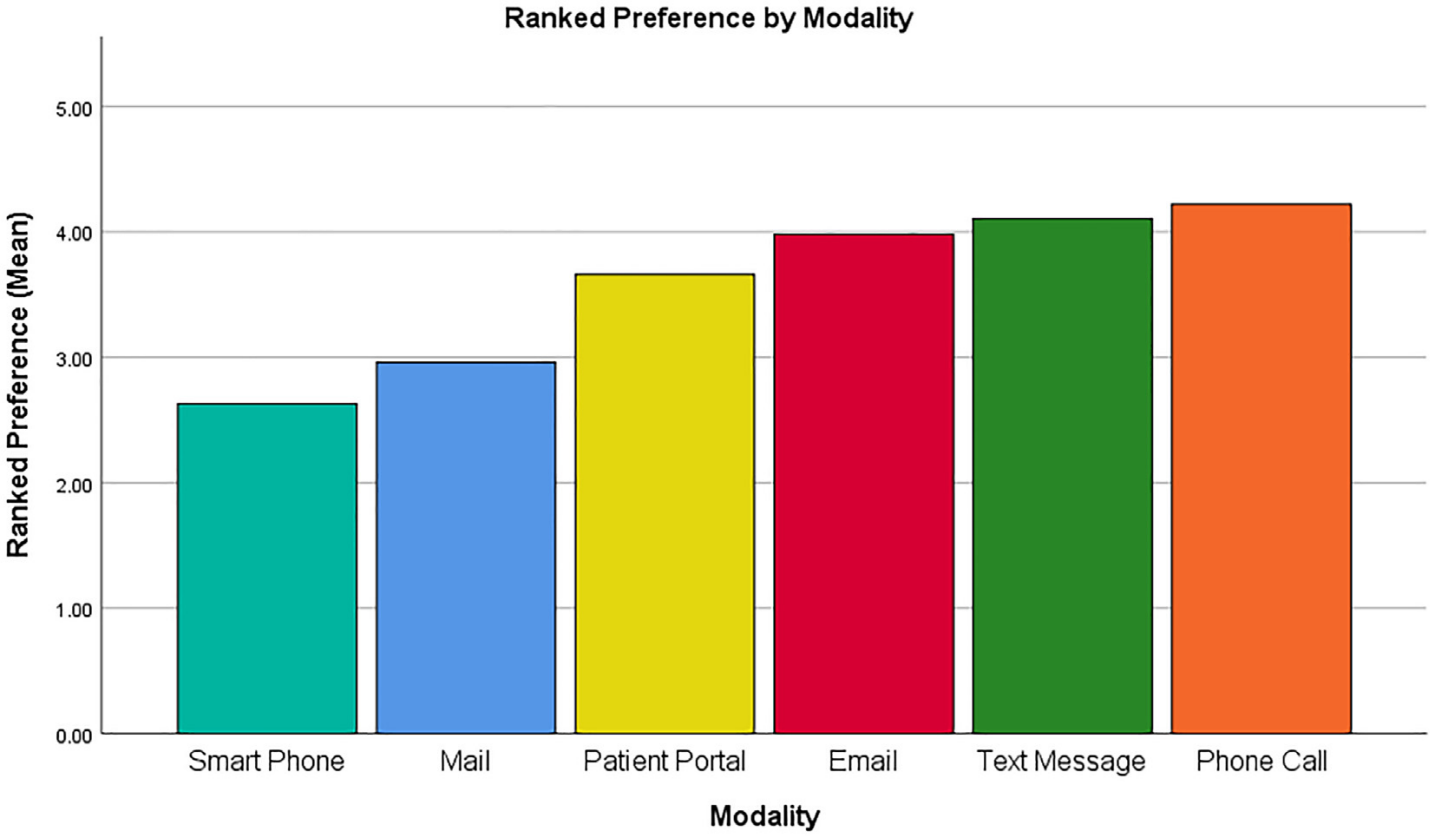
Mean patient-reported ranking of each treatment reminder system modality (1.00 = lowest ranking 5.00 = highest ranking).

The highest mean patient comfort level for phone calls was 4.2 (very comfortable) while the lowest, 3.2 (comfortable), was indicated for smartphone application (*p* < 0.05) (Fig. 1). The mean ranking for reminder system modality was significantly higher for phone call and text-message compared to smartphone application (*p* < 0.05) (Fig. 2). No correlation was found between preferred reminder system modality and type of incontinence, age, gender, race/ethnicity, or language spoken (*p* > 0.05). Internet usage and access significantly correlated with preference for smartphone applications and patient-portal message reminder systems (*p* < 0.05).

## 4 Discussion and conclusion

### 4.1 Discussion

This patient-centered qualitative study was designed to assess patients' perspectives on the use of treatment reminder systems to aid in the management and treatment adherence of UI. It was found that there was a significantly greater preference for phone call and text messaging compared to all other reminder system modalities such as email, patient portal, and smartphone applications.

Although no correlation was observed between patient demographics and reminder system preference, Peleg et al*,* identified that the accessibility and feasibility of mobile communication (phone calls and text messaging) across various demographics is considerably higher than other modalities (email and smartphone applications) [33]. Patient portal access was neither disfavored nor favored in this patient population. Although patient portals can be accessed on computers and mobile devices, it has been found that non-English speakers, racial and ethnic minorities, and people without commercial insurance are less likely to receive access to and become repeat users of a patient portal [34]. These differences may be due to socioeconomic barriers, cultural factors, or limitations in access to and awareness of the patient portal. Increased patient involvement in the management of their treatment plan has been found to improve outcomes, better understanding of these barriers and preferences could improve patient care and promote treatment adherence with the use of patient portals [35].

Smartphone application as a modality for a treatment reminder system was considerably disfavored by this patient population. Interestingly, numerous studies found the utility of mobile health apps in various patient populations [[19], [20], [21], [22]]. Buller et al. found that using a mobile app (REQ-Mobile) as an intervention was more effective than a control group of SMS text messaging for smoking abstinence [36]. While the advent of smartphone applications in various consumer settings is vast, it may not be as applicable to all healthcare settings and populations. Patient preferences should be taken into consideration before utilizing these resources and implementing patient care protocols. More specifically, it has been shown in this study that the role of smartphone applications as a reminder tool for conservative treatment of UI may be minimal in this population. Additionally, privacy, security, and patient preferences/barriers should be considered when selecting the most suitable system to help promote treatment adherence.

We also acknowledge that there is a complex interplay of other physical, emotional, and social factors related to UI, which can differ from one cohort to another. For the future, it would be important to explore these factors with respect to patients' satisfaction with their treatment progress and if there is a correlation with treatment reminder system choice. Additionally, the utility of treatment reminder systems needs to be further explored as it may contribute to decreasing healthcare burden by promoting at-home treatment adherence versus in-office discussions to promote treatment adherence. The cost-effectiveness and long-term effects of these reminder modalities should also be determined to further understand healthcare implications of these systems.

There are several limitations to the current study. A selection bias exists since not all patients seen at our clinic during the study period agreed to complete the survey. Although 90 patients were approached for the study, 33 declined to participate for reasons including disinterest in participating in research and/or lack of time during clinic visit, Also, since the surveys were anonymous without any identifying information, we were unable to contact a portion of the patients if certain variables were not answered in the survey. Sampling error and bias are risks of any survey in which not all subjects respond. The study also had a small sample size. Further larger studies are required to substantiate these findings and understand the utility of these reminders on treatment adherence and outcomes. Furthermore, the survey used in this study was not pilot tested, which would help further substantiate our findings. The failure to recruit individuals who are non-English speaking decreases the generalizability of this study. Finally, the qualitative rating scale on reminder system preference has not been validated with other objective measures, and is solely elicited from the patient's perspective, and no control group existed.

### 4.2 Innovation

The present study provides useful information regarding treatment reminder system preference in patients with UI demonstrating the valuable and potential use of readily accessible technologies for promoting treatment adherence in these patients. Specifically, this study demonstrated the potential utility of text messaging and phone call reminder modalities for patients with UI seeking help with treatment adherence.

### 4.3 Conclusion

This study demonstrated that patients with UI had a preference for text messaging and phone call for treatment reminder modalities and were found to be least comfortable with a smartphone application. Comfort with Internet use correlated with preference of smartphone application and patient-portal messaging. The next step is to develop automated reminder systems for patients based on their preference and assess treatment adherence.

## Funding

This research did not receive any specific grant from funding agencies in the public, commercial, or not-for-profit sectors.

## Research support

This research received no external financial or non-financial support.

## Relationships

There are no additional relationships to disclose.

## Patents and intellectual property

There no patents to disclose.

## Other activities

There are no additional activities to disclose.

## Declaration of Competing Interest

None.
